# Influence of Dextran Molecular Weight on the Physical Properties of Magnetic Nanoparticles for Hyperthermia and MRI Applications

**DOI:** 10.3390/nano10122468

**Published:** 2020-12-09

**Authors:** Oliver Strbak, Iryna Antal, Iryna Khmara, Martina Koneracka, Martina Kubovcikova, Vlasta Zavisova, Matus Molcan, Alena Jurikova, Petra Hnilicova, Jan Gombos, Nina Kadasova, Dusan Dobrota

**Affiliations:** 1 Biomedical Centre Martin, Jessenius Faculty of Medicine in Martin, Comenius University in Bratislava, Mala Hora 4, 036 01 Martin, Slovakia; petra.hnilicova@uniba.sk; 2 Institute of Experimental Physics, Slovak Academy of Sciences, Watsonova 47, 040 01 Kosice, Slovakia; iryna.antal@saske.sk (I.A.); irynakhmara@gmail.com (I.K.); kubovcikova@saske.sk (M.K.); zavisova@saske.sk (V.Z.); molcan@saske.sk (M.M.); akasard@saske.sk (A.J.); 3 Department of Medical Biochemistry, Jessenius Faculty of Medicine in Martin, Comenius University in Bratislava, Mala Hora 4, 036 01 Martin, Slovakia; gombos9@uniba.sk (J.G.); dusan.dobrota@uniba.sk (D.D.); 4 Department of Biophysics, Faculty of Science, Palacky University Olomouc, Krizkovskeho 511/8, CZ-779 00 Olomouc, Czech Republic; nina.kadasova@gmail.com

**Keywords:** magnetic fluid, magnetic nanoparticles, dextran, physical properties, diameter, magnetic hyperthermia, MRI, relaxivity

## Abstract

Dextran-coated magnetic nanoparticles are promising biocompatible agents in various biomedical applications, including hyperthermia and magnetic resonance imaging (MRI). However, the influence of dextran molecular weight on the physical properties of dextran-coated magnetic nanoparticles has not been described sufficiently. We synthesise magnetite nanoparticles with a dextran coating using a co-precipitation method and study their physical properties as a function of dextran molecular weight. Several different methods are used to determine the size distribution of the particles, including microscopy, dynamic light scattering, differential centrifugal sedimentation and magnetic measurements. The size of the dextran-coated particles increases with increasing dextran molecular weight. We find that the molecular weight of dextran has a significant effect on the particle size, efficiency, magnetic properties and specific absorption rate. Magnetic hyperthermia measurements show that heating is faster for dextran-coated particles with higher molecular weight. The different molecular weights of the coating also significantly affected its MRI relaxation properties, especially the transversal relaxivity *r_2_*. Linear regression analysis reveals a statistically significant dependence of *r_2_* on the differential centrifugal sedimentation diameter. This allows the targeted preparation of dextran-coated magnetic nanoparticles with the desired MRI properties. These results will aid the development of functionalised magnetic nanoparticles for hyperthermia and MRI applications.

## 1. Introduction

Biocompatible magnetic nanoparticles (MNPs) are essential in the field of nanomedicine as they provide non-toxic systems for various biomedical applications, including targeted drug delivery, magnetic hyperthermia (MH) and magnetic resonance imaging (MRI) [1,2,3,4,5]. MH is a promising therapeutic method in cancer treatment and is currently attracting increasing attention [6]. It includes an artificially induced increase in temperature (up to 45 °C) from the heat generated by the MNPs when subjected to an alternating magnetic field (AMF) [6]. MNPs possess a permanent magnetic moment. Through the application of an AMF, the energy needed for the reorientation of this magnetic moment is dissipated and converted to heat due to the relaxation and hysteresis processes [7,8]. Efforts have been focused on maximising the heat generation into the surrounding environment and this can be achieved by varying the applied magnetic field intensity and frequency or by increasing the concentration of MNPs [9]. Although increasing the MNP concentration is a simple approach, it is counterproductive regarding the later elimination of particles from the human body [10]. In the ideal situation, a small particle concentration can achieve high heating in a relatively short time. The term specific absorption rate (SAR) more precisely defines this relationship as the amount of heat released by a unit weight of the material per unit time during exposure to an AMF of defined frequency (*f*) and field strength (*H*) [11]. Apart from the application parameters (*H* and *f*), the SAR also depends on the magnetic component characteristics, such as particle size, distribution, shape and magnetic properties of MNPs, including coating/surfactant properties, which affect the hydrodynamic size and carrier liquid viscosity [5,12,13,14].

For biomedical applications, MNPs are usually coated with small molecules, such as amino acids [15,16] and surfactants [17,18], but can also be covered with larger polymers, such as poly-l-lysine [19,20,21], polyethylene glycol [22,23,24], poly(d,l-lactic acid) [25,26], polyvinyl alcohol [22,27], chitosan [28,29] and dextran (DEX) [22,30]. The main importance of coatings lies in ensuring colloidal and physical stability and biocompatibility. The effect of the coatings on the therapeutic efficiency must be considered and carefully studied. It has been shown that coatings increase SAR values in comparison with uncoated particles [20,31]. However, the efficacy of MH may also be affected by the molecular weight (MW) of the coating [20]. Jozefczak et al. [32] studied the influence of the MW of polyethylene glycol on the properties of biocompatible MNPs, where the heating effect was insignificant but the rheological properties were changed obviously. Siposova et al. [30] showed the impact of DEX MW for DEX-coated MNPs on amyloid aggregation. DEX is a polysaccharide produced by bacteria that is commonly used in a variety of biomedical applications [33]. Linh et al. [34] analysed DEX-coated MNPs of different concentrations for hyperthermia application, but the influence of MW on the SAR has not been studied.

Stabilised MNPs have also found use in MRI applications, especially in contrast enhancement [35]. Depending on the size and coating of the MNPs, they can accumulate in pathological sites or different tissue structures, increasing the contrast between physiological and pathological tissue, or different structures in the tissue [36]. DEX-coated MNPs have attracted significant attention due to their distinct MRI relaxivity properties, overall tissue distribution and excellent biocompatibility [37]. However, there is currently no information on the effect of DEX MW on the MRI contrast properties of DEX-stabilised MNPs, which could be a key factor for potential biomedical applications.

Therefore, in this study, we examine the influence of the MW of DEX in DEX-coated MNPs on the morphological and physical properties of the resulting biocompatible magnetic fluids (MFs) with respect to their application in hyperthermia and MRI. In our case, the MF is a colloidal suspension that consists of DEX-coated MNPs and a liquid carrier. The effect on the hydrodynamic diameter (*D*_HYDR_) and its resulting impact on the SAR and MRI relaxivity values are determined and explained. For this purpose, DEX-coated MNPs with three different MWs (40, 70 and 150 kDa) are prepared and analysed in MH and MRI experiments. The physical properties of the DEX-coated MNPs are studied using dynamic light scattering (DLS), differential centrifugal sedimentation (DCS), thermogravimetric analysis (TGA), transmission and scanning electron microscopy (TEM and SEM, respectively) and magnetic measurements.

## 2. Materials and Methods

### 2.1. Chemicals

All used chemicals are analytical graded without further purification. Ferric chloride hexahydrate (FeCl_3_•6H_2_O), ferrous chloride tetrahydrate (FeCl_2_•4H_2_O) and ammonium hydroxide (NH_4_OH) were purchased from Sigma-Aldrich (St. Louis, MO, USA). Dextran (MW = 40 and 70 kDa) were purchased from the AppliChem (Darmstadt, Germany) and Dextran with MW = 150 kDa was obtained from VWR (Radnor, PA, USA). In all experiments, deionized water was used.

### 2.2. Preparation of DEX-Coated MNPs

The DEX-coated MNPs were prepared using a slightly modified Molday procedure [30,38]. Briefly, 0.76 g of DEX (MW = 40, 70 or 150 kDa) was dissolved in 5 mL of water. Masses of 0.2 g of FeCl_3_ × 6H_2_O and 0.084 g of FeCl_2_ × 4H_2_O were dissolved in 0.5 mL of 2 M HCl. The reacting mixture consisting of ferrous and ferric chloride with DEX was placed into a thermomixer and heated to 60 °C under mixing. Then, 3 mL of 7.5% ammonium hydroxide were added dropwise and the formed nanoparticles were mixed for another 60 min at 60 °C.

### 2.3. Determination of Magnetite Concentration

The Fe_3_O_4_ concentration of each colloid was measured through ultraviolet spectrophotometry after completing the dissolution of the MNPs in acidic media. Ferrous ions present in the solution were oxidised to ferric ions by H_2_SO_4 (conc)_ and H_2_O_2_ prior to reacting with thiocyanate salt to form the iron-thiocyanate complex ([Fe(SCN)_6_]^3−^_(aq)_). A 50 µL aliquot of each DEX-MF was completely dissolved in 1:1 *v/v* H_2_SO_4 (conc)_ and H_2_O_2_ (1%) for 1.5 h at 60 °C. These solutions were then diluted in 50 mL flasks with distilled water. To the Fe^3+^ solutions, 1 mL of 1 M ammonium thiocyanate solution was added, and the iron concentration was determined by spectrophotometric measurements at 478 nm using a SPECORD^®^ 40 spectrophotometer (Analytik Jena AG, Jena, Germany) after 15 min [39].

### 2.4. Characterisation of DEX-MFs

For the determination of the hydrodynamic particle size (*D*_HYDR_), DLS measurements were performed using a Zetasizer (Nano ZS Nanoseries, Malvern Instruments, Malvern, UK) with a 4 mW He−Ne laser source (λ = 633 nm) operating in backscattering mode at an angle of 173°. This method enables to measure intensity weighted distribution, where the contribution of each particle in the distribution relates to the intensity of light scattered by the particle. The dispersions were diluted to obtain an optimal intensity of ~10^5^ counts per second. The zeta potential of the DEX-MFs was measured at 25 ± 0.1 °C using the same Zetasizer (Malvern Instruments Ltd., Malvern, UK) with disposable zeta cells (DTS 1060). The zeta standard of Malvern (−42 ± 4.2 mV) was used for calibration. The pH range and ionic strength were identical to those in the DLS experiments. The Smoluchowski equation was applied to convert the electrophoretic mobility to the zeta potential value. The accuracy of the measurements was ±5 mV.

The complementary technique used to determine the size distribution of the DEX-MFs was DCS. The DC24000 UHR disk centrifuge (CPS Instruments, Inc., Prairieville, LA, USA) was used to perform sedimentation based on size distribution measurements.

Using SEM (JEOL 7000F, Tokyo, Japan), the shape and particle size of the DEX-MFs were determined. A droplet of the water-diluted colloidal dispersion was deposited on a metal sample stub and dried under vacuum prior to sputtering with carbon and subsequent observation by SEM. For diameter determination, ~500 individual nanoparticles were analysed, and the resulting size distribution was fitted with a log-normal function.

TEM (Philips Tecnai 20, 200 kV, LaB6, FEI company, Hilsboro, Oregon, USA) was used to observe the MNPs of the studied samples. The samples were prepared by deposition of a 10 µL sample containing MNPs of diluted DEX-MFs on a carbon-coated copper support grid, followed by air drying.

Concerning the presence of the organic compound on the surface of the iron nanoparticles, TGA was performed on the dried samples under flowing air with a heating rate of 10 °C/min in a temperature range from 20 to 800 °C using a Differntial Thermal and Thermogravimetric Analyser (TGDTA SETSYS 16, Setaram, France). For this purpose, the samples of DEX-MFs were centrifuged at 93,000 rpm for 3 h, and the supernatant containing unbound DEX was removed. The sediment containing DEX-coated MNPs was then washed with distilled water and freeze-dried in a lyophiliser (ilShinBioBase Europe B.V., Ede, The Netherlands) at −52 °C.

A vibrating sample magnetometer installed on a cryogen-free superconducting magnet from Cryogenic Ltd. (Cryogenic Limited, London, UK) was used to investigate the magnetic properties of the samples. The measurements were performed in the zero-field cooling (ZFC) and field cooling (FC) regimes in the available temperature range from 5 to 300 K. For field-dependent magnetisation measurements, a magnetic field within ±5 T was applied.

An AMF was applied to study the influence of MW on the heating properties of the DEX-MFs. The thermally insulated sample (1 mL with a concentration 25 mg∙mL^−1^) in a glass vial was inserted into the cavity of a single-layer copper solenoid [40]. The temperature changes were recorded using a fibre optic temperature sensor (FISO Technologies, Québec, QC, Canada) with an accuracy of 0.1 K.

### 2.5. MRI

The MRI measurements were performed using a 7 T BioSpec Bruker system (Billerica, MA, USA). We used two different protocols for the determination of the longitudinal *T*_1_ and transversal *T*_2_ relaxation times:

- *T*_1_ mapping—rapid acquisition with refocused echoes (RARE) pulse sequence, with repetition time TR = 5500, 3000, 1500, 800, 400 or 200 ms, and echo time TE = 7 ms.
- *T*_2_ mapping—multi-slice multi-echo (MSME) pulse sequence, with repetition time TR = 2000 ms, starting echo time TE = 8 ms, spacing = 8 ms and 25 images.

The DEX-coated MNPs were divided into three independent groups, based on the *D*_HYDR_, but with the same concentration:

- DEX_40_-MF: DEX-coated MNPs with a MW of DEX equal to 40 kDa and *D*_HYDR_ = 72.03 nm.
- DEX_70_-MF: DEX-coated MNPs with a MW of DEX equal to 70 kDa and *D*_HYDR_ = 88.37 nm.
- DEX_150_-MF: DEX-coated MNPs with a MW of DEX equal to 150 kDa and *D*_HYDR_ = 122.2 nm.

First, the signal intensity values were acquired and evaluated as the relative contrast (RC) values. The RC of the MNPs as a negative contrast agent (I_0_ > I) is defined as follows:

RC = (I − I_0_)/I_0_, (1)

where I_0_ is the signal intensity without magnetite particles, and I represents the signal intensity with magnetite nanoparticles.

Subsequently, we determined the longitudinal and transversal relaxation times (*T*_1_ and *T*_2_) of the DEX-coated MNPs by fitting with the following functions:

*M(t) = A + M*_0_*× (*1 − *exp(t/T*_1_*))*, (2)

*y = A + C × exp(−t/T*_2_*)*, (3)

where *M*_0_ is the equilibrium magnetisation, *A* is the absolute bias, *T*_1_ is the longitudinal recovery time, *C* is the signal intensity and *T*_2_ is the transversal relaxation time. Finally, we calculated and evaluated the transversal and longitudinal relaxation rates (*R*_1_ and *R*_2_) and relaxivity (*r*_1_ and *r*_2_) values. We used the concentration gradient of DEX-coated magnetite nanoparticles (2.5–15 µg mL^−1^) for the relaxivity *r* evaluation. The transversal relaxation rate (*R_n_*) is inversely related to the relaxation time (*T_n_*):

*R_n_ =* 1*/T_n_* (*n* = 1 or 2) (4)

The change in *R_n_*, which characterises the efficiency of magnetic particles for contrast properties in MRI, is defined as the relaxivity of the particle (contrast agent):

*r_n_ = (R_n_ − R_n_^0^)/C_MNPs_* (*n* = 1 or 2) (5)

where R*_n_*^0^ is the relaxation rate in the absence of magnetic particles, *R_n_* represents the relaxation rate in the presence of magnetic particles and *C*_MNPs_ is the magnetic particle concentration. In our samples, we determined the relaxivity value using a linear fit of the relaxation rate *R* dependence on the molar concentration of iron (0–0.2 mM).

We employed Paravision image sequence analysis (Bruker, Billerica, MA, USA) and Matlab R2019a software (Mathworks Inc., Natick, MA, USA) for MRI data processing.

## 3. Results and Discussion

### 3.1. Physical Characterisation of DEX-Coated MNPs

**Figure 1:**
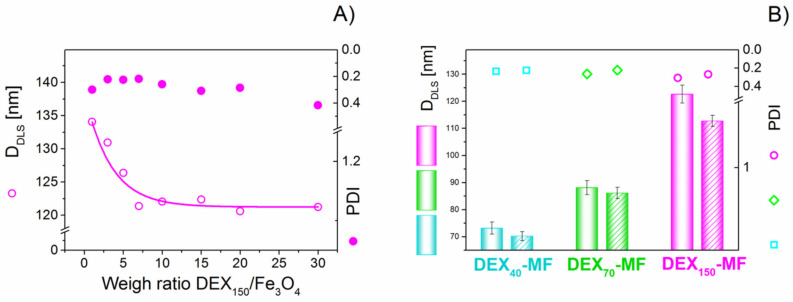
Effects of DEX_150_ /Fe_3_O_4_ ratio in the samples of DEX_150_–magnetic fluids (MFs) on D_HYDR_ and polydispersity index (PDI) (**A**); average D_HYDR_ and PDI of magnetite nanoparticles in the samples of DEX*_x_*-MFs at 20 °C determined by dynamic light scattering (DLS) (**B**).

A layer of biodegradable polymer changes the *D*_HYDR_ of MNPs [19,24,41,42,43]. A series of tested samples with a constant concentration of magnetite and different amounts of DEX_150_ (DEX_150_/Fe_3_O_4_ feed weight ratios from 1 to 30) was prepared to determine the optimal conditions for the DEX adsorption on MNPs. The results of the measurements of the particle size are shown in Figure 1. The size of the nanoparticles decreases with the amount of DEX added to the MNP reaction mixture, reaching stabilisation at a weight ratio of DEX_150_/Fe_3_O_4_ from 6 to 30. This can be explained by the fact that more DEX in the reaction mixture prevents agglomeration and sedimentation of the MNPs [44]. For a DEX_150_/Fe_3_O_4_ ratio range from 1 to 20, the polydispersity index (PDI) varies from 0.218 to 0.309. For the ratio of DEX_150_/Fe_3_O_4_ equal to 30, PDI reaches a maximum value of 0.416, indicating a broader size distribution. Based on these results, the optimal weight ratio of DEX adsorption on the MNPs was 6. This weight ratio was chosen for the fabrication of the DEX_40_-MF, DEX_70_-MF and DEX_150_-MF samples.

For the biomedical application of MNPs, a mean particle diameter of <200 nm and a narrow size distribution are desirable to avoid the risk of embolism. Therefore, a knowledge of particle size is an essential requirement. The prepared DEX*_x_*-MFs (*x* = 40, 70 or 150 kDa) were studied by four different sizing methods (DLS, DCS, SEM and TEM) to determine the mean size and the size distribution of particles.

DLS is sensitive to dynamic aggregation, aggregation, agglomeration and so on. Furthermore, the measurement of sizes from DLS data is an indirect method, based on the determination of the frequency of movement and modelling of the size from this data. Thus, there are several reasons to expect different results from this technique compared to the microscopic techniques. The intensity distributions of DEX*_x_*-MFs are shown in Figure 1B. As shown by the figure, the *D*_HYDR_ of the DEX*_x_*-coated nanoparticles becomes larger with increasing DEX MW. This result is in accordance with previous reports [24,45]. The PDI varies from 0.233 to 0.308. All prepared nanoparticle suspensions show stability against sedimentation for 20 months (Figure 1B shaded columns). The hydrodynamic sizes of these samples are almost unchanged. The same equipment was used to measure the zeta potential of all DEX*_x_*-MFs. The zeta potential is a parameter that helps to characterise the surface of nanoparticles. If an efficient coverage of MNPs by DEX is achieved, it is reasonable to predict that the value of the zeta potential is changed. As seen in Table 1, in all measured samples, the zeta potential decreased in comparison with the zeta potential of the naked MNPs (~18 mV). Moreover, the DEX*_x_*-MFs display an isoelectric point (IEP) lower than the IEP of the naked MNPs, suggesting that DEX was adsorbed on the MNPs.

DLS and DCS are used for quantitative particle size distribution analysis of samples in the submicron region. DCS is a high-resolution particle sizing technique that utilises Stokes’ law to estimate an unknown particle size distribution in a known centrifugal field by measuring the sedimentation time of the particles in a fluid of known density and viscosity. As seen in Table 1, a slightly increasing particle size with increasing DEX MW used for particle modification was measured.

**Figure 2:**
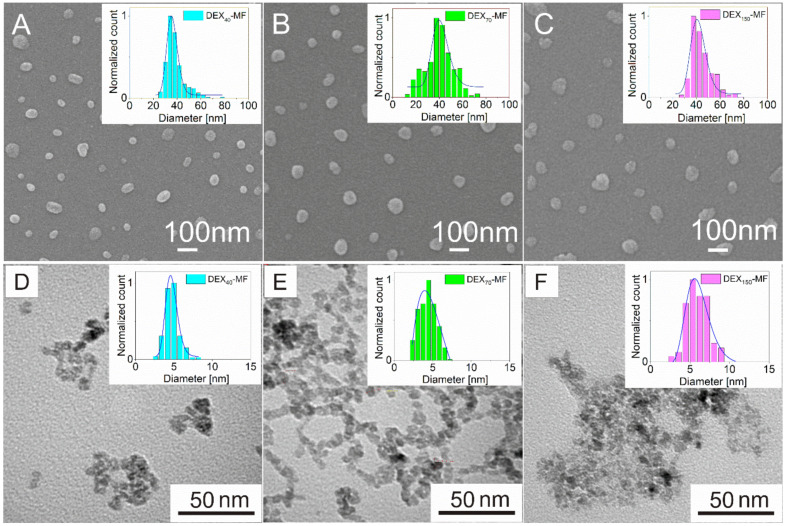
SEM images of DEX_40_-MF (**A**), DEX_70_-MF (**B**) and DEX_150_-MF (**C**) samples and corresponding size distributions. TEM images of MNPs in DEX_40_-MF (**D**), DEX_70_-MF (**E**) and DEX_150_-MF (**F**) samples and corresponding size distributions.

A third method is related to microscopic evaluation. SEM allows us to observe the particles and evaluate their range of shapes and sizes. Particles in the obtained SEM and TEM images were measured manually, i.e., INSCAPE software was used to draw circles or ellipses the edge of the particles. From the obtained data and recalculation, histograms of the size distribution were plotted. From the measured data, a log-normal distribution is derived. Figure 2A–C shows the morphological characterisation of the DEX_40_-MF, DEX_70_-MF and DEX_150_-MF nanoparticles obtained by SEM with the corresponding histograms showing the particle size distributions. The studied nanoparticles in the samples were roughly spherical in shape with a smooth surface. The particle size slightly increases with the DEX MW (Figure 2 and Table 1). The value of mean nanoparticle diameter (*D*_SEM_) determined from the log-normal fit of the histogram increased from 35.8 (DEX_40_-MF) to 41.5 nm (DEX_150_-MF). These values are in good agreement with the diameter obtained by DCS measurements.

The final method used to measure the particle size and morphology is TEM. TEM provides accurate mean particle size analysis compared to the previously mentioned methods as they include a few coating layers. Figure 2D–F displays the TEM images of the DEX*_x_*-MF samples measured by the previous sizing methods. While SEM creates an image by detecting reflected or knocked-off electrons, TEM uses transmitted electrons (electrons that pass through the sample) to create an image. As a result, TEM offers valuable information on the inner (magnetite core) structure of the sample. It was found that the magnetic particles of the DEX*_x_*-MFs had irregularly shaped cores with a size range of ~3–8 nm, with nanoparticles forming chains or clusters of several cores. In the DEX_150_-MF sample, it is most evident that the particles are coated with DEX, even completely submerged in it, which also resulted in a reduced quality of focus. The magnetic mean core sizes determined from the log-normal fit of the histogram were equal to 4.7 nm (Standard Deviation, SD = 0.9 nm), 4.3 nm (SD = 1.1 nm) and 5.7 nm (SD = 1.5 nm) for DEX_40_-MF, DEX_70_-MF and DEX_150_-MF, respectively.

It is very likely that the main differences between the measured diameters from these four techniques are due to the presence of an adsorbing layer, which is composed of DEX on the surface of the magnetic particles. Molecular size organic compounds, such as DEX, are electron transparent, and therefore did not appear in the TEM micrograph.

### 3.2. Stability of DEX-Coated MNPs

**Figure 3:**
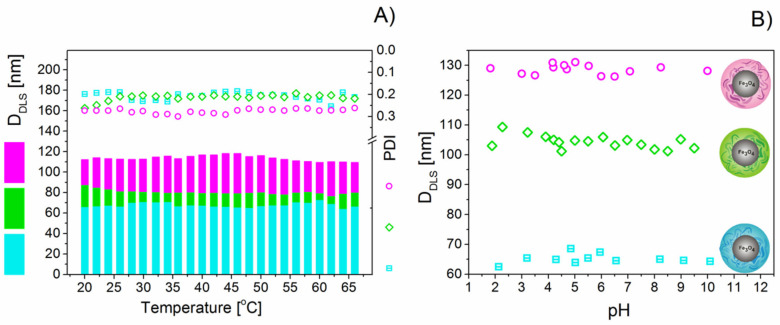
Evolution of the average *D*_HYDR_ and PDI in DEX*_x_*-MF samples as a function of temperature (**A**) and pH (**B**).

The stability of the DEX*_x_*-MF samples was monitored using the Zetasizer Nano ZS by monitoring their *D*_HYDR_ as a function of temperature and pH. As seen in Figure 3A, the changes in the average *D*_HYDR_ of the DEX*_x_*-MFs are plotted as a function of temperature. DLS measurements were conducted at temperatures ranging from 20 to 65 °C at a 2 °C step and allowing temperature stabilisation for a period of 2 min before each measurement. No change in the *D*_HYDR_ of the particles was observed between 20 and 65 °C, confirming the thermal stability of the DEX*_x_*-MFs. In terms of colloidal stability, all DEX*_x_*-MFs showed very good stability against pH changes in the measured region. No significant changes in the *D*_HYDR_ of the DEX*_x_*-MFs were observed in the whole measured pH range (Figure 3B).

### 3.3. Thermogravimetric Analysis of DEX-Coated MNPs

**Figure 4:**
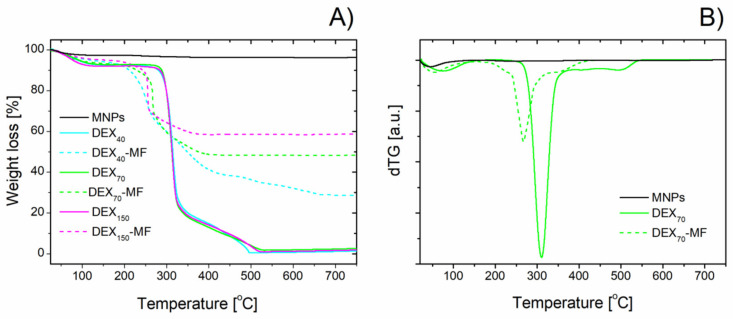
TG curves for samples of magnetite, DEX*_x_* and DEX*_x_*-MFs (**A**) and the derivative TG curves for magnetite, DEX_70_ and DEX_70_-MF (**B**).

Representative results from the TGA measurements can be seen in Figure 4. Decomposition thermograms for the studied samples of magnetite, DEX*_x_* and DEX*_x_*-MF are presented in Figure 4A. To better distinguish the decomposition processes, the derivatives of the thermograms for DEX_70_ and DEX_70_-MF are shown in Figure 4B.

Bare magnetite is stable in the whole measured temperature range except for a small initial weight loss of 3.6% due to residual water evaporation. The decomposition of free DEX*_x_* goes through three stages during the heating up to 800 °C, almost independent of the DEX MW. In the first stage, only a small weight loss up to 150 °C, related to the water evolution of DEX (~8%), was observed. The second decomposition stage with a significant weight loss of ~75% occurs in the temperature range of ~250–370 °C and is associated with the breakdown of the organic skeleton of DEX. The decomposition (characterised with a 15% weight loss) then continues up to 530 °C, at which DEX is almost completely decomposed.

Similar decomposition stages were revealed for the DEX-coated MNPs (DEX*_x_*-MFs); however, their thermal behaviour is different for the different MWs of DEX. The initial small weight loss (~6%) up to 150 °C due to dehydration is followed by a much more significant process that finished at ~330–350 °C with weight losses of 44%, 41% and 35% for *x* = 40, 70 and 150, respectively. This decomposition process can be associated with the oxidative degradation of DEX [46]. The next DEX decomposition up to 430–450 °C was only accompanied by weight losses of 11%, 4% and 2% for *x* = 40, 70 and 150, respectively, and according to [46] was caused by the elimination of carbonaceous residue deposited on the magnetite surface.

It can be concluded that the two-step thermal decomposition of bound DEX (from 150 to 430 °C) is shifted to lower temperatures in comparison to free DEX (from 250 to 530 °C) by ~100 °C. This effect is observed for the samples coated with DEX of all different MWs and can be associated with the catalytic effect of the magnetite on the DEX decomposition in the DEX*_x_*-MF samples [46,47].

Taking into account that all DEX is decomposed at 750 °C and the magnetite shows thermal stability in the measured temperature range, the difference between the remaining weight of the magnetite and DEX-coated samples at this temperature can be related to the amount of DEX bound on the MNPs. As seen in Figure 4A, this amount strongly depends on the DEX MW. At 750 °C, the DEX_40_-MF, DEX_70_-MF and DEX_150_-MF samples exhibited remaining weights of 28.6%, 48.4% and 58.9%, respectively. Considering the obtained TGA results, the amounts of DEX bound on the MNPs were found to be 2.37, 0.99 and 0.64 mg DEX*_x_*/mg Fe_3_O_4_ for *x* = 40, 70 and 150, respectively. The higher the MW, the lower the amount of DEX observed. The decrease in the absorbed DEX amount with increasing MW is in accordance with the results reported for other modifying polymers [48,49].

### 3.4. Magnetic Properties of DEX-Coated MNPs

Among the many applications of MNPs, those in biomedicine are particularly interesting and widespread. Generally, the magnetic properties required for these applications are superparamagnetic behaviour at room temperature, high saturation magnetisation and sizes in the 1−50 nm range. In addition, biocompatibility and functionalisation requirements are imposed to coat the nanoparticles with a protective layer that may modify the magnetic properties of the core nanoparticles.

**Figure 5:**
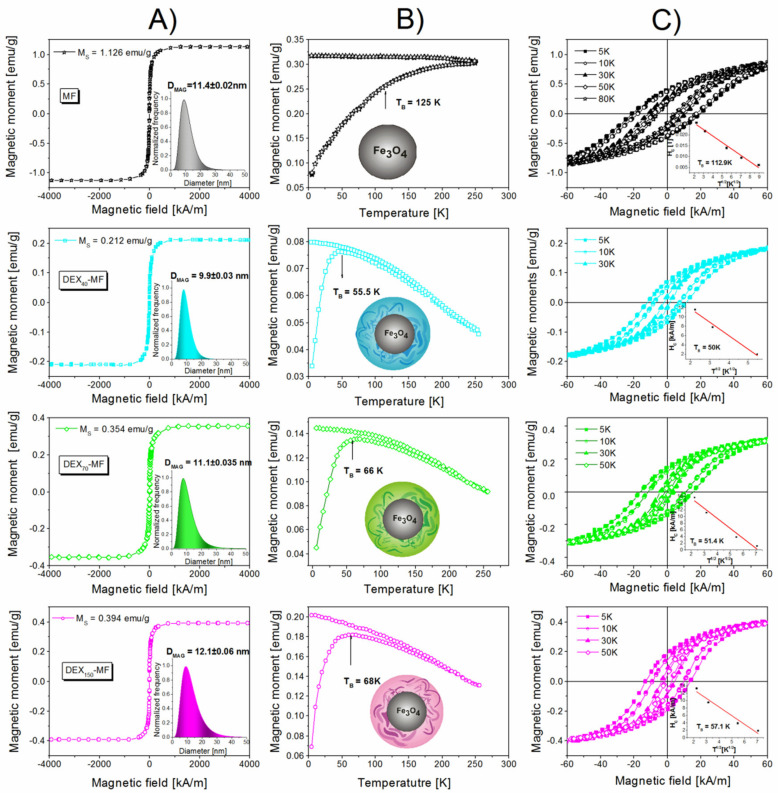
Hysteresis loops of samples measured at 298 K with insets representing magnetic size distributions (**A**); magnetic moment versus temperature for the MF and DEX*_x_*-MFs at 100 Oe at zero field and field cooling (**B**); field dependence of magnetic moment at different temperatures below *T_B_*. Insets show coercivity (*H_C_*) plot with respect to *T*^1/2^ (**C**).

One of the key aims in the work was to study the magnetic properties of naked and DEX (with different MWs)-coated MNPs. The results of the saturation magnetisation analysis can be seen in Figure 5A. No hysteresis is observed, and the magnetisation curves are completely reversible, exhibiting the superparamagnetic behaviour of the prepared samples. The saturation magnetisation (*M*_S_) values from the magnetisation curves were found to be 1.13 emu∙g^−1^ for naked MNPs, 0.21 emu∙g^−1^ for DEX_40_-MF, 0.35 emu∙g^−1^ for DEX_70_-MF and 0.39 emu∙g^−1^ for DEX_150_-MF. The coating of magnetite nanoparticles with DEX reduces the *M*_S_ values. This reduction is in direct relation to the DEX coating amount on the MNPs obtained from TGA (see Table 1).

The blocking temperature (*T*_B_) is a crucial factor that must be addressed in the case of biomedical applications because of the importance of the superparamagnetic behaviour of the particles. Above *T*_B_, the nanoparticles do not magnetically interact due to the randomisation of their magnetisation, whereas below *T*_B_, the particles interact and offer a ferromagnetic-like behaviour. To facilitate the utilisation of the particles in biomedicine, *T*_B_ must be below room temperature to guarantee the disappearance of the remanence when the magnetic field is switched off. Therefore, FC/ZFC measurements were made to investigate *T*_B_. Figure 5B shows the FC and ZFC curves for the MF and DEX*_x_*-MFs in the range of 5 to 300 K at 100 Oe. The *T_B_* of the magnetic moment is indicated by the maximum in the ZFC curve, but the exact determination of *T_B_* was calculated from the derivation of the ZFC curve where the derivate curve intercepts the x axis. The calculated *T_B_* values are summarised in Table 1. Moreover, the *T_B_* values provide information regarding the magnetic interaction, i.e., there is a general trend where *T_B_* shifts towards higher temperatures when the strength of the interaction between particles increases. The decrease in the *T_B_* value of the DEX*_x_*-MFs in comparison with the *T_B_* of the naked MNPs indicates that the average distance between the magnetic particles significantly changes, thereby confirming the DEX layer on the MNP surface.

For further understanding of the magnetic properties of the DEX-coated MNPs, we performed magnetic hysteresis loop measurements below the *T_B_* as it is known in the theory of superparamagnetism that the coercivity of the sample has a temperature dependence below the blocking temperature, as follows [50]:

(6) $$\frac{H}{H_{C0}}=1-\sqrt{\frac{T}{T_{B}}}$$

where *H*_C0_ is the coercivity at 0 K. The insets in Figure 5C show the coercivity with respect to *T*^1/2^. The *T_B_* was obtained from the least squares fitting of the temperature-dependent coercivity. It is noteworthy that the *T_B_* values obtained from the magnetic hysteresis measurements as a function of temperature are in good agreement with the FC/ZFC measurements results (see Table 1).

### 3.5. Magnetic Hyperthermia of DEX-Coated MNPs

**Figure 6:**
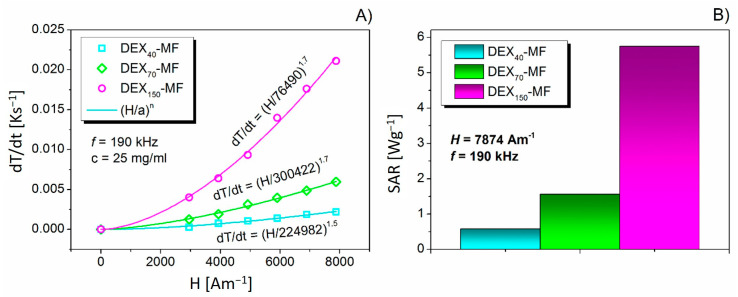
Dependence of *dT/dt* on the magnetic field amplitude *H* for the DEX*_x_*-MFs (with MNPs concentration 25 mg∙mL^−1^) and the function of the fit (**A**); Specific absorption rate evolution for DEX*_x_*-MF samples (**B**).

An AMF with the six used field intensities *H* (between 3 and 8 kA∙m^−1^) and a frequency of *f* = 190 kHz was applied for a period of 90 s for all DEX*_x_*-MF (with *x* = 40, 70 and 150 kDa) samples. Temperature evolution dependence curves in time (not presented) were obtained on this basis. *ΔT* was very significant for the sample with MW = 150 kDa (1.7 °C/90 s). In contrast, for the samples with smaller MWs, it was just tenths of °C for the same period (90 s). *T* versus *t* curves were subsequently plotted by a linear fit. From this fitting, heating rates (*dT/dt*) at given *H* values were obtained. The *dT/dt* versus *H* dependencies were subsequently fitted by a (*H/a)^n^* function [51], where *a* and *n* are fitting parameters. If the sample includes only superparamagnetic nanoparticles (as proven by the magnetisation measurements), *dT/dt = (H/a)^n^* is a square function and *n* ≅ 2 [52]. Figure 6A shows the dependence of *dT/dt* on the magnetic field amplitude *H* for the DEX*_x_*-MFs and the function of the fit. In our case, the obtained value of the *n* parameter shows that losses occur through the thermal energy associated with the magnetic relaxation. Although the heating is faster for the samples with higher MW, the power law is approximately the same (*n*~1.7) for all three samples. From this point of view, MW has no impact on the physical principle of the heating.

Ultimately, the MW affected the SAR values. The increase in MW leads to a higher release in thermal energy. The dependence of the SAR evolution on the MW is presented in Figure 6B. The SAR was estimated according to the following equation [52]:

(7) $$\mathrm{SAR}=\frac{c_{p}\rho_{S}}{m}\left(\frac{dT}{dt}\right)$$

where *C_p_* is the specific heat capacity (*C_p_ ≈ C_water_* = 4.18 J∙K^−1^∙g^−1^), *ρ*_S_ is the density of the sample (≈1021 kg∙m^−3^), *m* is the mass of magnetite per unit volume of the colloid (25 mg∙mL^−1^) and *dT/dt* is the heating rate (slope) calculated from the linear fit of the heating curves. In the case of smaller MWs (DEX_40_-MF and DEX_70_-MF), the SAR values are very low, but in the case of the DEX_150_-MF, the increase is obvious. This behaviour is due to magnetic particles with longer DEX molecules on the surface, which do not track the changes in the magnetic field and thus generate higher energy losses compared to magnetite nanoparticles with shorter DEX chains.

### 3.6. MRI Relaxivity Determination of DEX-Coated MNPs

We measured the MRI relaxivity properties of DEX-coated MNPs with two different relaxation time mapping protocols (RARE—*T*_1_ and MSME—*T*_2_). Longitudinal *T*_1_ and transversal *T*_2_ relaxation times were determined and analysed. The same concentration gradient (2.5–15 µg/mL) of the MNPs was used to calculate the longitudinal *r*_1_ and transversal *r*_2_ relaxivities of DEX-coated MNPs with different MWs of DEX. As the relaxivity r characterises the sensitivity of the material to MRI contrast, the aim is to determine whether different MWs of the DEX coating and the resulting different diameters affect the relaxivity value. DEX itself does not affect the MRI signal (data not presented).

**Figure 7:**
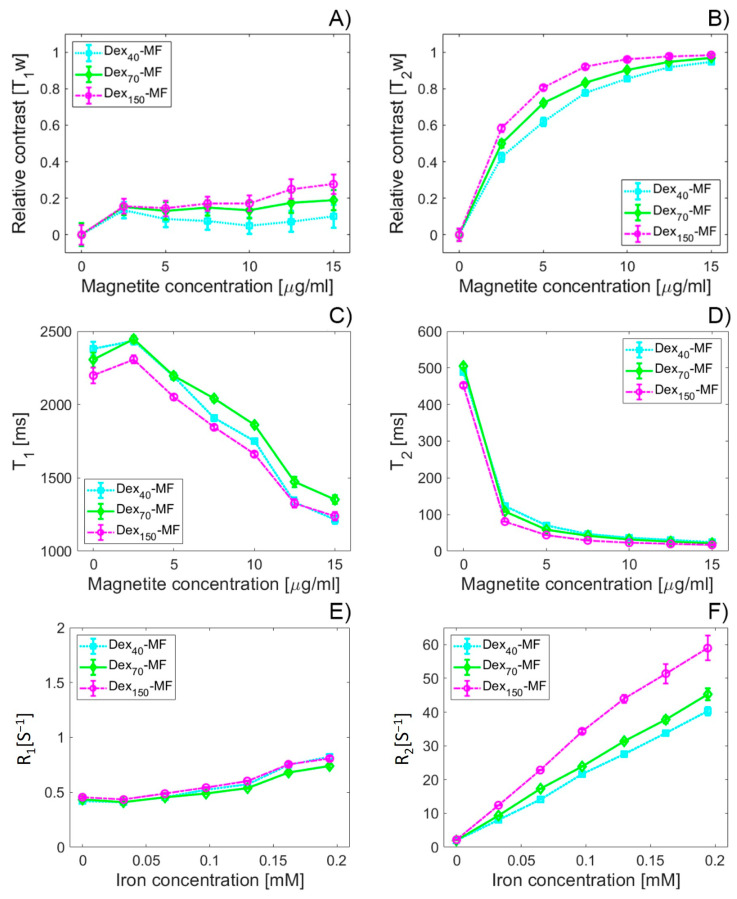
RC of DEX-coated MNPs with different MWs of DEX coating. RC of the *T*_1_-weighted protocol (**A**); RC of the *T*_2_-weighted protocol (**B**); relaxation time of the DEX-coated MNPs with different MWs of DEX coating, longitudinal relaxation time *T*_1_ (**C**), transversal relaxation time *T*_2_ (**D**); relaxation rate of the DEX-coated MNPs with different MWs of DEX coating, longitudinal relaxation rate *R*_1_ (**E**); transversal relaxation rate *R*_2_ (**F**).

MNPs are generally described as negative contrast agents, which means that they primarily affect the transversal relaxation time *T*_2_ in comparison with the longitudinal relaxation time *T*_1_, as shown in Figure 7 and Figures S1–S4 in Supplementary Materials. The RC of the *T*_1_-weighted sequence varies from 0.05 to 0.28 (Figure 7A), while the RC values from the T_2_-weighted sequence are from 0.425 to 0.98 (Figure 7B). This clearly indicates the prevailing negative contrast of the DEX-coated MNPs and the emergence of the so-called hypointensive artefacts in the images. In both cases, the increase in the MW of DEX mimics the increase in RC (RC_DEX40-MF_ < RC_DEX70-MF_ < RC_DEX150-MF_). Only in the case of the highest concentration of magnetite in the *T*_2_-weighted sequence and the lowest concentration in the *T*_1_-weighted protocol do the individual values almost overlap (Figure 7).

**Figure 8:**
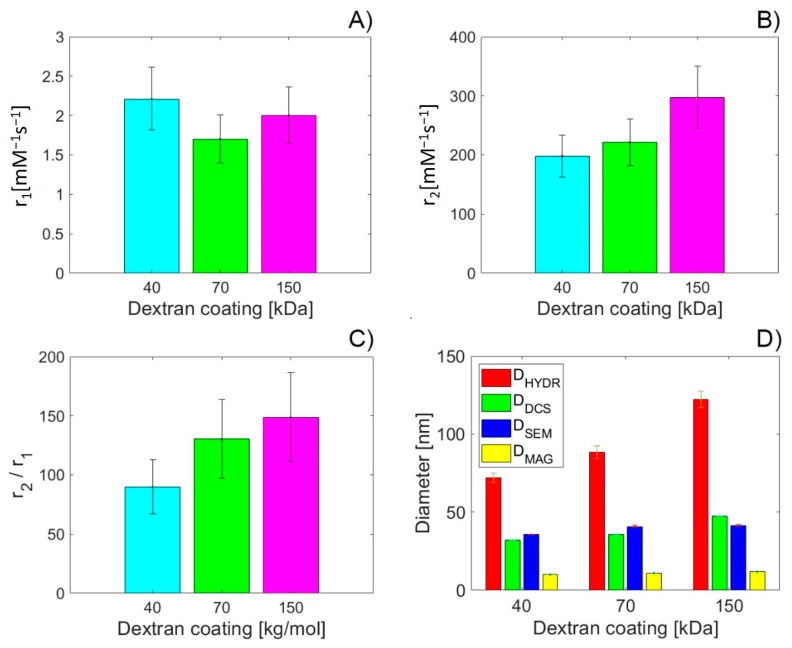
Relaxivity of the DEX-coated MNPs with different MWs of DEX coating. Longitudinal relaxivity *r*_1_ (**A**); transversal relaxivity *r*_2_ (**B**). Transversal *r*_2_ and longitudinal relaxivity *r*_1_ ratio of the DEX-coated MNPs with different MWs of DEX coating (**C**). Diameter of the DEX-coated MNPs with different MW of DEX coating (**D**).

Surprisingly, the situation in Figure 7A is not identical with the image in Figure 7C, which shows the T_1_ relaxation time itself. While the initial increase in the value of the T_1_ relaxation time copies the initial slight increase in the RC of all three samples, the further course of the graphs is different. Shortening of the longitudinal relaxation time T_1_ in all coatings follows the mentioned initial increase, which has been observed previously for low magnetite concentrations [20]. As magnetite is a typical negative contrast agent, a significant decrease in the T_2_ relaxation time (Figure 7D) was expected and copies the behaviour in Figure 8B. Relaxation rate plots, both for the longitudinal *R*_1_ (Figure 7E) and transversal R_2_ rates (Figure 7F), were used to determine the relaxivity value for all samples (see Supplementary Materials, Figures S5 and S6).

The longitudinal *r*_1_ and transversal r_2_ relaxivity values of the DEX-coated MNPs with different MWs of DEX coating are shown in Figure 8A,B. Both values were obtained through linear fitting of the longitudinal and transverse relaxation rates, *R*_1_ and *R*_2_, as shown in the supplementary material (Figures S5 and S6). Longitudinal relaxivity *r*_1_ is from 1.7 to 2.6 mM^−1^ s^−1^ (Figure 8A), while transversal relaxation is from 198.1 up to 297.1 mM^−1^ s^−1^ (Figure 8B). For all tested DEX MWs, the transversal relaxivity *r*_2_ is higher than the transversal relaxivity value of the commercially available magnetite-based contrast agent (Resovist, *r*_2_ = 189 mM^−1^ s^−1^ [53]). In comparison with the study of Mishra et al. [54], who also studied the DEX-coated magnetite nanoparticles prepared by co-precipitation method and with a size of 12 nm (determined by TEM), our MNPs show lower longitudinal relaxivity *r*_1_ (Mishra et al.: *r*_1_ = 18.4 ± 0.3 mM^−1^ s^−1^) and higher transversal relaxivity *r*_2_ (Mishra et al.: *r*_2_ = 90.5 ± 0.8 mM^−1^ s^−1^) for all studied DEX MWs. A key feature when comparing the contrast efficacy of the T_2_ contrast agents is the transversal and longitudinal relaxivity ratio *r*_2_/*r*_1_. The higher the *r*_2_/*r*_1_ ratio, the better the contrast efficacy [55]. The *r*_2_/*r*_1_ ratio of our samples at 7 T is in the range of 89.7 to 148.6 mM^−1^ s^−1^ (Figure 8C), which is 18–30 times more in comparison with the findings of Mishra et al. [54].

In addition to determining the relaxivity of the DEX-coated MNPs with various MWs of DEX, we focused on the possible correlation and causality of the relaxivity values on the diameter, determined by various methods: DLS, DCS, SEM and magnetic measurements (Figure 8D). Figure S7 in Supplementary Materials shows the correlation coefficients between the relaxivity values and diameters obtained by different techniques. A strong positive correlation is observed for transversal relaxivity *r*_2_, as well as for the *r*_2_/*r*_1_ ratio. In addition, a weak to modest negative correlation is observed for longitudinal relaxivity *r*_1_. Based on previously published data [54], such a relationship was expected and confirms the T_2_ contrast properties of the DEX-coated MNPs exclusively.

Regarding the causality of the relaxivity values and the various types of diameter, we performed linear regression analysis. Figure S8 in Supplementary Materials compares the linear fit slopes of all diameters established by different measurement techniques, with the slopes of relaxivity values *r*_2_, and *r*_2_/*r*_1_, for all studied MWs of DEX (40, 70 and 150 kDa). Surprisingly, the linear slopes of the *D*_HYDR_ and *r*_2_/*r*_1_ ratio are almost identical. The results of the linear regression analysis for longitudinal and transversal relaxivity, as well as for their ratio, are shown in Table 2 and Figures S9–S11 in Supplementary Materials. Based on the goodness-of-fit statistics from Table 2, it is obvious that the best fit has been achieved for transversal relaxivity r_2_ dependence on the *D*_DCS_. In this case, the result allows the targeted preparation of DEX-coated MNPs with a specific desired transversal relaxivity value *r*_2_, only by the *D*_DCS_ modification. A similar result, but with the greater deviation of the values from the fit (SSE) and standard error of the regression (RMSE), was observed for the transversal relaxivity r_2_ dependence on the *D*_HYDR_ (Figure S10A) and the *D*_MAG_ (Figure S10D).

For the *r*_2_/*r*_1_ ratio dependence on the diameter, the statistically acceptable, but again with greater SSE and RMSE values, are dependencies on the *D*_SEM_ and *D*_MAG_ (Figure S11C,D). In other cases, the statistical significance of the fit is much lower (Figure S10C and Figure S11A,B). Moreover, in the case of the longitudinal relaxivity *r*_1_, it is negligible for all studied diameters (Figure S9). These results confirm the exclusive nature of DEX-coated MNPs as a *T*_2_ contrast agent and reveal the impact of different MWs of the DEX-coating on the resulting diameter and subsequently on the transversal relaxivity *r*_2_ and *r*_2_/*r*_1_ ratio, respectively.

## 4. Conclusions

DEX-coated MNPs have attracted significant attention due to their distinctive MRI relaxivity properties, overall tissue distribution and excellent biocompatibility. However, there was a lack of information regarding the influence of the MW of DEX coating on the physical and MRI properties of such modified MNPs. We have found that the MW of DEX has a significant effect on the size of modified MNPs, coating efficiency, magnetic properties and the SAR.

The optimal weight ratio of the DEX adsorption on MNPs was found to be 6. With increasing MW of DEX, the particle size increased, which was confirmed by five different methods. Furthermore, with increasing MW, the saturation magnetization of the samples also increased due to the decrease in the amount of DEX adsorbed per unit mass of MNP. In addition, magnetic measurements confirmed superparamagnetic behaviour in all prepared samples. TGA outcomes showed that the adsorbed DEX amount in the DEX*_x_*-MFs decreased with increasing MW of the loaded DEX. These results are in coincidence with the data from magnetic saturation. MH measurements of the DEX*_x_*-MF showed that the heating is faster for the samples with higher MW, as the increase in MW leads to the release of higher thermal energy compared to MNPs with shorter DEX chain. The SAR value was almost ten times higher in the DEX_150_-coated MNPs compared to the DEX_40_-coated MNPs. However, the SAR values do not reach the applicability of the relaxivity values in MRI applications. In our samples, the SAR may be increased by the increased coating, which in turn could negatively affect the MRI relaxation properties. Therefore, this issue deserves closer attention to the next study.

MRI results confirm the exclusive nature of DEX-coated MNPs as a *T_2_* contrast agent and reveal the significant impact of different MWs of the DEX coating on the resulting diameter and subsequently on the transversal relaxivity *r_2_* and *r_2_/r_1_* ratio, respectively. Moreover, linear regression analysis showed that the statistically most significant model, regarding the influence of various diameters of DEX-coated MNPs on MRI relaxivity, is transversal relaxivity *r_2_* dependence on *D*_DCS_ diameter (*R^2^ =* 100%). This allows for the targeted preparation of DEX-coated MNPs with a specific desired transversal relaxivity value *r_2_*, only by the *D*_DCS_ modification. We believe that these results could have a significant impact on the development and preparation of novel DEX-coated MNPs, designed for hyperthermia and MRI-related biomedical applications.

## Figures and Tables

**Table 1:** Physicochemical properties of DEX*_x_*-MF obtained by different techniques.

Sample	Adsorbed DEX*_x_* [mg DEX/ mg Fe_3_O_4_]	ζ [mV]	*D*_DCS_ [nm]	*D*_SEM_ [nm]	*M*_S_ [emu/g]	*D*_MAG_ [nm]	*T*_B_ * [K]	*T*_B_ ** [K]	IEP	SAR [W/g]
DEX_40_-MF	2.37	−0.7 ± 0.17	32.3	35.8 ± 7.8	0.212	10.1 ± 0.1	56	49	5.2	0.6
DEX_70_-MF	0.99	−1 ± 0.133	35.8	40.9 ± 11.7	0.354	11.1 ± 0.1	66	51	5.0	1.6
DEX_150_-MF	0.64	−1.3 ± 0.4	47.4	41.5 ± 8.6	0.394	12.0 ± 0.2	68	57	5.5	5.9

Amount of adsorbed DEX*_x_* on magnetic nanoparticles (MNPs) determined from TGA, ζ-potential—electrokinetic potential, *D*_DCS_—particle diameter determined from DCS measurements, *D*_SEM_—particle diameter determined from SEM images, *Ms*—saturation magnetisation, *D*_MAG_—core diameter determined from magnetic measurements, *T*_B_ *—blocking temperature determined from ZFC/FC measurement, *T*_B_ **—blocking temperature determined from the coercivity dependence with respect to *T*^1/2^, IEP—isoelectric point, SAR—specific absorption rate.

**Table 2:** Parameters of the linear regression of the relaxivity dependence on the diameter of DEX-coated MNPs determined by various techniques.

Sample	*b*	*a*	*SSE*	*R*^2^ [%]	adj-*R*^2^ [%]	*RMSE*
*r*_1_						
*D*_HYDR_	−0.002	2.179	0.1249	4.91	−90	0.3535
*D*_DCS_	−0.004	2.122	0.1295	1.47	−97	0.36
*D*_SEM_	−0.06	4.39	0.057	56.42	12.83	0.2393
*D*_MAG_	−0.12	3.275	0.11	19.11	−61.78	0.326
*r*_2_						
*D*_HYDR_	2.01	49.36	50.38	99.06	98.12	7.1
*D*_DCS_	6.55	−13.09	0.112	100	100	0.3335
*D*_SEM_	12.38	−248.9	2346	56.18	12.36	48.43
*D*_MAG_	51.56	−331.7	549.6	89.73	79.47	23.44
*r*_2_/*r*_1_						
*D*_HYDR_	1.08	21.24	295	83.77	67.55	17.18
*D*_DCS_	3.32	−4.89	443.2	75.63	51.26	21.05
*D*_SEM_	9.41	−248	80.28	95.59	91.17	8.96
*D*_MAG_	31.18	−222.2	61.92	96.59	93.19	7.87

*a*, *b*—coefficients of the linear fit *y* = *bx* + *a*, with 95% confidence bounds; *SSE*—sum of square errors; *R*^2^—square of the multiple correlation coefficient; adj-*R*^2^—adjusted *R*^2^; *RMSE*—root mean square error.

## Supplementary Materials

The following are available online at https://www.mdpi.com/2079-4991/10/12/2468/s1. Figure S1: *T*_1_-weighted MRI images of DEX-coated MNPs at different concentration of magnetite (from top left: 0, 0, 2.5, 5, 7.5, 10, 15 µg/mL of magnetite). Dextran coating: 40 kDa (A); 70 kDa (B); 150 kDa (C); Figure S2: *T*_2_-weighted MRI images of DEX-coated MNPs at different concentration of magnetite (from top left: 0, 0, 2.5, 5, 7.5, 10, 15 µg/mL of magnetite). Dextran coating: 40 kDa (A); 70 kDa (B); 150 kDa (C); Figure S3: *T*_1_ mapping of DEX-coated MNPs at different concentration of magnetite (from top left: 0, 0, 2.5, 5, 7.5, 10, 15 µg/mL of magnetite). Dextran coating: 40 kDa (A); 70 kDa (B); 150 kDa (C); Figure S4: *T*_2_ mapping of DEX-coated MNPs at different concentration of magnetite (from top left: 0, 0, 2.5, 5, 7.5, 10, 15 µg/mL of magnetite). Dextran coating: 40 kDa (A); 70 kDa (B); 150 kDa (C); Figure S5: Longitudinal relaxivity *r*_1_ determination using a linear fit of the longitudinal relaxation rate *R* dependence on the iron concentration. Dextran coating of DEX-coated MNPs: 40 kDa (A); 70 kDa (B); 150 kDa (C); Figure S6: Transversal relaxivity *r*_2_ determination using a linear fit of the transversal relaxation rate *R* dependence on the iron concentration. Dextran coating of DEX-coated MNPs: 40 kDa (A); 70 kDa (B); 150 kDa (C); Figure S7: Correlation coefficients between relaxivity values and diameter of DEX-coated MNPs, determined by various techniques: *D*_HYDR_ (A), *D*_DCS_ (B), *D*_SEM_ (C), *D*_MAG_ (D); Figure S8: Comparison of the linear fit slopes of all diameters established by different measurement techniques—*D*_HYDR_, *D*_DCS_, *D*_SEM_ and *D*_MAG_, with transversal relaxivity *r*_2_ and relaxivity ratio *r*_2_/*r*_1_ (A); Course of the D_HYDR_ and *r*_2_/*r*_1_ curves of the DEX-coated MNPs with different MWs of DEX coating (B); Figure S9: Linear regression analysis of the longitudinal relaxivity *r*_1_ dependence on the diameter of DEX-coated MNPs determined by various techniques. *D*_HYDR_ (A), *D*_DCS_ (B), *D*_SEM_ (C), *D*_MAG_ (D); Figure S10: Linear regression analysis of the transversal relaxivity *r*_2_ dependence on the diameter of DEX-coated MNPs determined by various techniques: *D*_HYDR_ (A), *D*_DCS_ (B), *D*_SEM_ (C), *D*_MAG_ (D); Figure S11: Linear regression analysis of the *r*_2_/*r*_1_ ratio dependence on the diameter of DEX-coated MNPs determined by various techniques: *D*_HYDR_ (A), *D*_DCS_ (B), *D*_SEM_ (C), *D*_MAG_ (D).

## References

[1] Colombo M., Carregal-Romero S., Casula M.F., Gutiérrez L., Morales M.P., Böhm I.B., Heverhagen J.T., Prosperi D., Parak W.J. (2012). Biological applications of magnetic nanoparticles. Chem. Soc. Rev..

[2] Varanda L.C., Júnior M.J., Júnior W.B., Laskovski A.N. (2011). Magnetic and Multifunctional Magnetic Nanoparticles in Nanomedicine: Challenges and Trends in Synthesis and Surface Engineering for Diagnostic and Therapy Applications. Biomedical Engineering.

[3] Kashkooli F.M., Soltani M., Souri M. (2020). Controlled anti-cancer drug release through advanced nano-drug delivery systems: Static and dynamic targeting strategies. J. Control. Release.

[4] Hola K., Markova Z., Zoppellaro G., Tucek J., Zboril R. (2015). Tailored functionalisation of iron oxide nanoparticles for MRI, drug delivery, magnetic separation and immobilisation of biosubstances. Biotechnol. Adv..

[5] Das P., Colombo M., Prosperi D. (2019). Recent advances in magnetic fluid hyperthermia for cancer therapy. Colloids Surf. B Biointerfaces.

[6] Liu X., Zhang Y., Wang Y., Zhu W., Li G., Ma X., Zhang Y., Shizu C., Tiwari S., Shi K. (2020). Comprehensive understanding of magnetic hyperthermia for improving antitumor therapeutic efficacy. Theranostic.

[7] Dutz S., Hergt R. (2014). Magnetic particle hyperthermia—A promising tumour therapy?. Nanotechnology.

[8] Molcan M., Gojzewski H., Skumiel A., Dutz S., Kovac J., Kubovcikova M., Kopcansky P., Vekas L., Timko M. (2016). Energy losses in mechanically modified bacterial magnetosomes. J. Phys. D Appl. Phys..

[9] Ortega D., Pankhurst Q.A., O’Brien P. (2013). Magnetic Hyperthermia, in Nanoscience: Volume 1: Nanostructures through Chemistry.

[10] Frtus A., Smolkova B., Uzhytchak M., Lunova M., Jirsa M., Kubinova S., Dejneka A., Lunov O. (2020). Analysing the mechanisms of iron oxide nanoparticles interactions with cells: A road from failure to success in clinical applications. J. Control. Release.

[11] Lanier O.L., Korotych O.I., Monsalve A.G., Wable D., Savliwala S., Grooms N.W.F., Nacea C., Tuitt O.R., Dobson J. (2019). Evaluation of magnetic nanoparticles for magnetic fluid hyperthermia. Inter. J. Hyperth..

[12] Babic M., Horak D., Molcan M., Timko M. (2017). Heat generation of surface-modified magnetic γ-Fe_2_O_3_ nanoparticles in applied alternating magnetic field. J. Phys. D Appl. Phys..

[13] Osaci M., Cacciola M. (2020). About the influence of the colloidal magnetic nanoparticles coating on the specific loss power in magnetic hyperthermia. J. Magn. Magn. Mater..

[14] Shaterabadi Z., Nabiyouni G., Soleymani M. (2020). Correlation between effects of the particle size and magnetic field strength on the magnetic hyperthermia efficiency of dextran-coated magnetite nanoparticles. Mater. Sci. Eng. C.

[15] Antal I., Koneracka M., Kubovcikova M., Zavisova V., Khmara I., Lucanska D., Jelenska L., Vidlickova I., Zatovicova M., Pastorekova S. (2018). D,L-Lysine functionalised Fe_3_O_4_ nanoparticles for detection of cancer cells. Colloids Surf. B Biointerfaces.

[16] Kaczmarek K., Hornowski T., Antal I., Timko M., Józefczak A. (2019). Magneto-ultrasonic heating with nanoparticles. J. Magn. Magn. Mater..

[17] Bica D., Vekas L., Avdeev M.V., Marinica O., Socoliuc V., Balasoiu M., Garamus V.M. (2007). Sterically stabilised water based magnetic fluids: Synthesis, structure and properties. J. Magn. Magn. Mater..

[18] Araújo-Neto R.P., Silva-Freitas E.L., Carvalho J.F., Pontes T.R.F., Silva K.L., Damasceno I.H.M., Egito E.S.T., Dantas A.L., Morales M.A., Carriço A.S. (2014). Monodisperse sodium oleate coated magnetite high susceptibility nanoparticles for hyperthermia applications. J. Magn. Magn. Mater..

[19] Khmara I., Koneracka M., Kubovcikova M., Zavisova V., Antal I., Csach K., Kopcansky P., Vidlickova I., Csaderova L., Pastorekova S. (2017). Preparation of poly-l-lysine functionalised magnetic nanoparticles and their influence on viability of cancer cells. J. Magn. Magn. Mater..

[20] Kubovcikova M., Koneracká M., Strbak O., Molcan M., Zavisova V., Antal I., Khmara I., Lucanska D., Tomco L., Barathova M. (2019). Poly-L-lysine designed magnetic nanoparticles for combined hyperthermia, magnetic resonance imaging and cancer cell detection. J. Magn. Magn. Mater..

[21] Babic M., Horak D., Trchova M., Jendelova P., Glogarova K., Lesna P., Herynek V., Hajek M., Sykova E. (2008). Poly(l-lysine)-modified iron oxide nanoparticles for stem cell labeling. Bioconjugate. Chem..

[22] Zavisova V., Koneracka M., Kovac J., Kubovcikova M., Antal I., Kopcansky P., Bednarikova M., Muckova M. (2015). The cytotoxicity of iron oxide nanoparticles with different modifications evaluated in vitro. J. Magn. Magn. Mater..

[23] Zavisova V., Koneracka M., Gabelova A., Svitkova B., Ursinyova M., Kubovcikova M., Antal I., Khmara I., Jurikova A., Molcan M. (2016). Effect of magnetic nanoparticles coating on cell proliferation and uptake. J. Magn. Magn. Mater..

[24] Kubovcikova M., Antal I., Koneracka M., Zavisova V., Jurikova A., Siposova K., Gazova Z., Kovac J., Kovarik M., Kupka D. (2013). Magnetic nanoparticles modified with polyethylene glycol. Magnetohydrodynamics.

[25] Antal I., Kubovcikova M., Zavisova V., Koneracka M., Pechanova O., Barta A., Cebova M., Antal V., Diko P., Zduriencikova M. (2015). Magnetic poly(d,l-lactide) nanoparticles loaded with aliskiren: A promising tool for hypertension treatment. J. Magn. Magn. Mater..

[26] Strbak O., Antal I., Gogola D., Baciak L., Kubovcikova M., Koneracka M., Zavisova V., Krafcik A., Masarova-Kozelova M., Kopcansky P. (2017). Measurement of the magnetite nanoparticles’ relaxivity during encapsulation into polylactide carriers. Measurement.

[27] Rahayu L.B.H., Wulandari I.O., Santjojo D.H., Sabarudin A. (2018). Synthesis and characterization of Fe_3_O_4_ nanoparticles using polyvinyl alcohol (PVA) as capping agent and glutaraldehyde (GA) as crosslinker. Mater. Sci. Eng..

[28] Khmara I., Strbak O., Zavisova V., Koneracka M., Kubovcikova M., Antal I., Kavecansky V., Lucanska D., Dobrota D., Kopcansky P. (2019). Chitosan-stabilized iron oxide nanoparticles for magnetic resonance imaging. J. Magn. Magn. Mater..

[29] Li G.Y., Jiang Y.R.,  Huang K.L., Ding P., Chen J. (2008). Preparation and properties of magnetic Fe_3_O_4_–chitosan nanoparticles. J. Alloys Compd..

[30] Siposova K., Pospiskova K., Bednarikova Z., Safarik I., Safarikova M., Kubovcikova M., Kopcansky P., Gazova Z. (2017). The molecular mass of dextran used to modify magnetite nanoparticles affects insulin amyloid aggregation. J. Magn. Magn. Mater..

[31] Majeed J., Pradhan L., Ningthoujam R.S., Vatsa R.K., Bahadur D., Tyagi A.K. (2014). Enhanced specific absorption rate in silanol functionalised Fe_3_O_4_ core–shell nanoparticles: Study of Fe leaching in Fe_3_O_4_ and hyperthermia in L929 and HeLa cells. Colloids Surf. B Biointerfaces.

[32] Jozefczak A., Hornowski T., Skumiel A., Zavisova V., Koneracka M., Tomasovicova N., Timko M., Kopcansky P., Kelani H.N. (2012). Effect of the molecular weight of poly(ethylene glycol) on the properties of biocompatible magnetic fluids. Int. J. Thermophys..

[33] Maia J., Evangelista M.B., Gil H., Ferreira L. (2014). Dextran-based materials for biomedical applications. Res. Signpost.

[34] Linh P.H., Phuc N.X., Hong L.V., Uyen L.L., Chien N.V., Nam P.H., Quy N.T., Nhung H.T.M., Phong P.T., Lee I.J. (2018). Dextran coated magnetite high susceptibility nanoparticles for hyperthermia applications. J. Magn. Magn. Mater..

[35] Wang Y.X., Hussain S.M., Krestin G.P. (2001). Superparamagnetic iron oxide contrast agents: Physicochemical characteristics and applications in MR imaging. Eur. Radiol..

[36] Portet D., Denizot B., Rump E., Lejeune J.J., Jallet P. (2001). Nonpolymeric coatings of iron oxide colloids for biological use as magnetic resonance imaging contrast agents. J. Colloid. Interface Sci..

[37] Hong R.Y., Feng B., Chen L.L., Liu G.H., Li Z.H., Zheng Y., Wei D.G. (2008). Synthesis, characterisation and MRI application of dextran-coated Fe_3_O_4_ magnetic nanoparticles. Biochem. Engin. J..

[38] Molday R.S., Mackenzie D. (1982). Immunospecific ferromagnetic iron-dextran reagents for the labeling and magnetic separation of cells. J. Immunol. Methods.

[39] Woods J., Mellon M. (1941). Thiocyanate Method for Iron: A Spectrophotometric Study. Determination of iron by thiocyanate colorimetry. Ind. Eng. Chem. Anal. Ed..

[40] Skumiel A., Kaczmarek K., Flak D., Rajnak M., Antal I., Brzakala H. (2020). The influence of magnetic nanoparticle concentration with dextran polymers in agar gel on heating efficiency in magnetic hyperthermia. J. Mol. Liq..

[41] Larsen E.K.U., Nielsen T., Wittenborn T., Rydtoft L.M., Lokanathan A.R., Hansen L., Ostergaard L., Kingshott P., Howard K.A., Besenbacher F. (2012). Accumulation of magnetic iron oxide nanoparticles coated with variably sized polyethylene glycol in murine tumors. Nanoscale.

[42] LaConte L.E.W., Nitin N., Zurkiya O., Caruntu D., O’Connor C.J., Hu X., Bao G. (2007). Coating thickness of magnetic iron oxide nanoparticles affects R_2_ relaxivity. J. Magn. Reason. Imaging.

[43] Xue W., Liu Y., Zhang N., Yao Y., Ma P., Wen H., Huang S., Luo Y., Fan H.M. (2018). Effects of core size and PEG coating layer of iron oxide nanoparticles on the distribution and metabolism in mice. Int. J. Nanomed..

[44] Shaterbadi Z., Nabiyouni G., Soleymani M. (2017). High impact of *in situ* dextran coating on biocompatibility, stability and magnetic properties of iron oxide nanoparticles. Mater. Sci. Eng. C.

[45] Sperling R.A., Liedl T., Duhr S., Kudera S., Zanella M., Lin C.A.J., Chang W.H., Braun D., Parak W.J. (2007). Size determination of (bio)conjugated water-soluble colloidal nanoparticles: A comparison of different techniques. J. Phys. Chem. C.

[46] Carp O., Patron L., Culita D.C., Budrugeac P., Feder M., Diamandescu L. (2010). Thermal analysis of two types of dextran-coated magnetite. J. Therm. Anal. Calorim..

[47] Koneracka M., Antosova A., Zavisova V., Lancz G., Gazova Z., Siposova K., Jurikova A., Csach K., Kovac J., Tomasovicova N. (2010). Characterization of Fe_3_O_4_ magnetic nanoparticles modified with dextran and investigation of their interaction with protein amyloid aggregates. Acta Phys. Polonica A.

[48] Butterworth M.D., Illum L., Davis S.S. (2001). Preparation of ultrafine silica- and PEG-coated magnetite particles. Colloids Surf. A Physicochem. Eng. Asp..

[49] Jurikova A., Csach K., Miskuf J., Koneracka M., Zavisova V., Kubovcikova M., Kopcansky P., Muckova M. (2013). Thermal properties of magnetic nanoparticles modified with polyethylene glycol. IEEE Trans. Magn..

[50] Yoona M., Kima Y.M., Kima Y., Volkova V., Songa H.J., Parkb Y.J., Vasilyakc S.L., Parka I.-W. (2003). Magnetic properties of iron nanoparticles in a polymer film. J. Magn. Magn. Mater..

[51] Khmara I., Molcan M., Antosova A., Bednarikova Z., Zavisova V., Kubovcikova M., Jurikova A., Girman V., Baranovicova E., Koneracka M. (2020). Bioactive properties of chitosan stabilised magnetic nanoparticles—Focus on hyperthermic and anti-amyloid activities. J. Magn. Magn. Mater..

[52] Skumiel A., Leszczyński B., Molcan M., Timko M. (2016). The comparison of magnetic circuits used in magnetic hyperthermia. J. Magn. Magn. Mater..

[53] Kostevsek N. (2020). A review on the optimal design of magnetic nanoparticle-based T_2_ MRI contrast agents. Magnetochemistry.

[54] Mishra S.K., Hemanth Kumar B.S., Khushu S., Tripathi R.P., Gangenahalli G. (2016). Increased transverse relaxivity in ultrasmall superparamagnetic iron oxide nanoparticles used as MRI contrast agent for biomedical imaging. Contrast Media Mol. Imaging.

[55] Qin J., Laurent S., Jo Y.S., Roch A., Mikhaylova M., Bhujwalla Z.M., Muller R.N., Muhammed M. (2007). A high-performance magnetic resonance imaging T_2_ contrast agent. Adv. Mater..

