# Supplementary material for: Influence of Dextran Molecular Weight on the Physical Properties of Magnetic Nanoparticles for Hyperthermia and MRI Applications
Source: Nanomaterials (Basel). 2020 Dec 9;10(12):2468. doi: 10.3390/nano10122468 (PMC7763203; doi:10.3390/nano10122468)
Supplement: Supplementary file 1 [file nanomaterials-10-02468-s001.pdf]

Supplementary Materials: Influence of Dextran Molecular Weight on the Physical Properties of Magnetic Nanoparticles for Hyperthermia and MRI Applications

Oliver Strbak [1,*], Iryna Antal [2], Iryna Khmara [2], Martina Koneracka [2,*], Martina Kubovcikova [2], Vlasta Zavisova [2], Matus Molcan [2], Alena Jurikova [2], Petra Hnilicova [1], Jan Gombos [3], Nina Kadasova [4] and Dusan Dobrota [3]

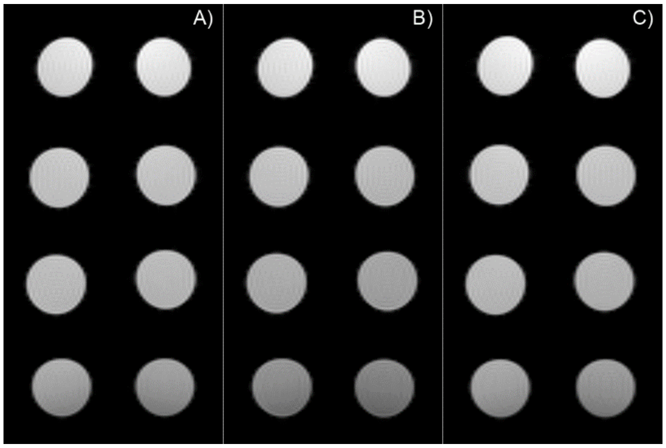

Figure S1. T_1-weighted MRI images of DEX-coated MNPs at different concentration of magnetite (from top left: 0, 0, 2.5, 5, 7.5, 10, 15 μg/mL of magnetite). Dextran coating: 40 kDa (**A**); 70 kDa (**B**); 150 kDa (**C**).

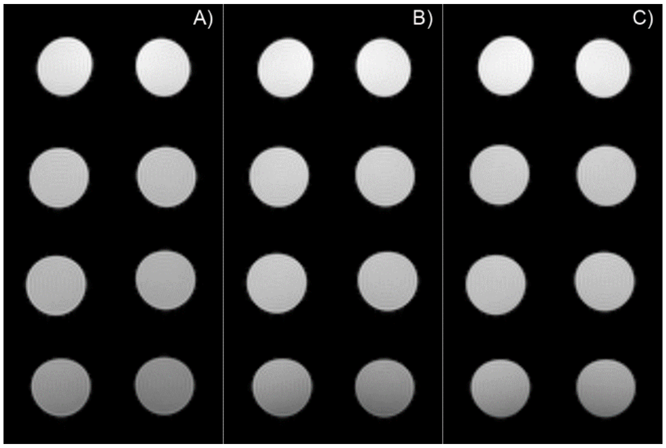

Figure S2. T_2-weighted MRI images of DEX-coated MNPs at different concentration of magnetite (from top left: 0, 0, 2.5, 5, 7.5, 10, 15 μg/mL of magnetite). Dextran coating: 40 kDa (**A**); 70 kDa (**B**); 150 kDa (**C**).

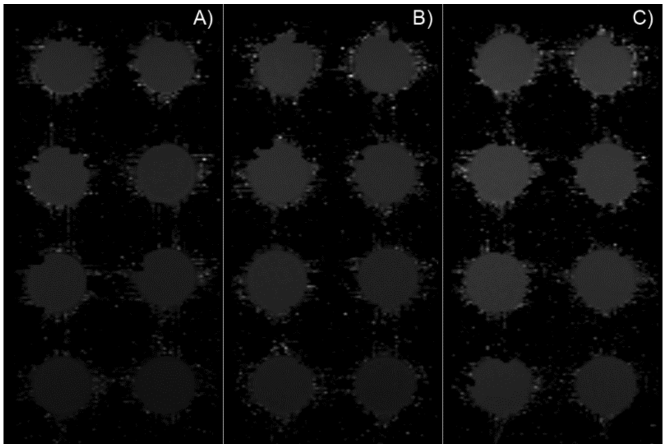

Figure S3. T_1 mapping of DEX-coated MNPs at different concentration of magnetite (from top left: 0, 0, 2.5, 5, 7.5, 10, 15 μg/mL of magnetite). Dextran coating: 40 kDa (**A**); 70 kDa (**B**); 150 kDa (**C**).

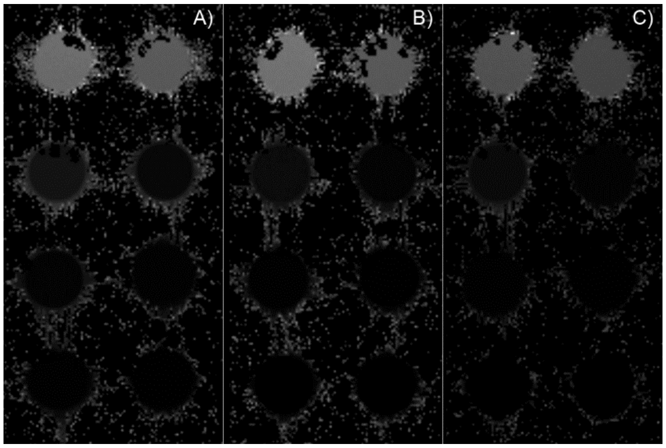

Figure S4. T_2 mapping of DEX-coated MNPs at different concentration of magnetite (from top left: 0, 0, 2.5, 5, 7.5, 10, 15 μg/mL of magnetite). Dextran coating: 40 kDa (**A**); 70 kDa (**B**); 150 kDa (**C**).

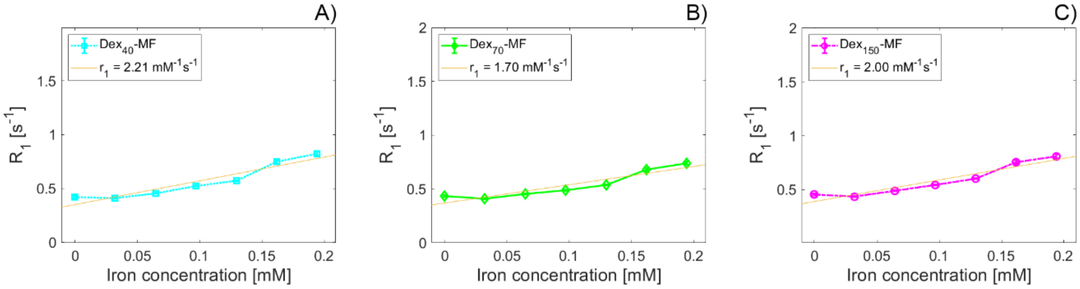

Figure S5. Longitudinal relaxivity r_1 determination using a linear fit of the longitudinal relaxation rate *R* dependence on the iron concentration. Dextran coating of DEX-coated MNPs: 40 kDa (**A**); 70 kDa (**B**); 150 kDa (**C**).

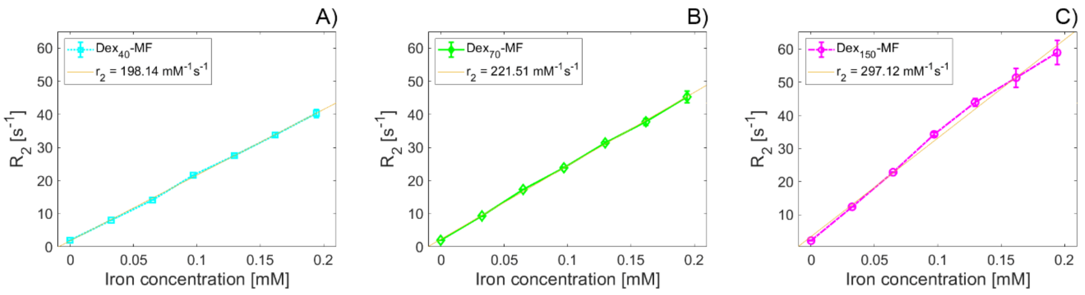

Figure S6. Transversal relaxivity r_2 determination using a linear fit of the transversal relaxation rate R dependence on the iron concentration. Dextran coating of DEX-coated MNPs: 40 kDa (**A**); 70 kDa (**B**); 150 kDa (**C**).

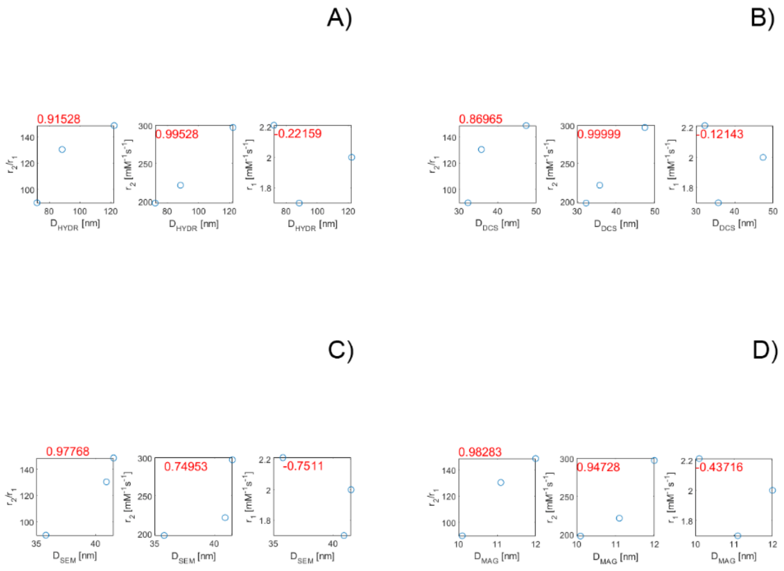

Figure S7. Correlation coefficients between relaxivity values and diameter of DEX-coated MNPs, determined by various techniques: D_{HYDR} (**A**), D_{DCS} (**B**), D_{SEM} (**C**), D_{MAG} (**D**)

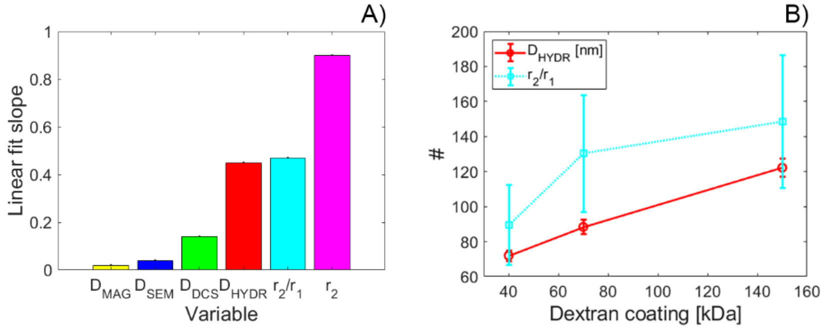

Figure S8. Comparison of the linear fit slopes of all diameters established by different measurement techniques - D_{HYDR}, D_{DCS}, D_{SEM} and D_{MAG}, with transversal relaxivity r_2 and relaxivity ratio r_2/r_1 (**A**); Course of the D_{HYDR} and r_2/r_1 curves of the DEX-coated MNPs with different MWs of DEX coating (**B**)

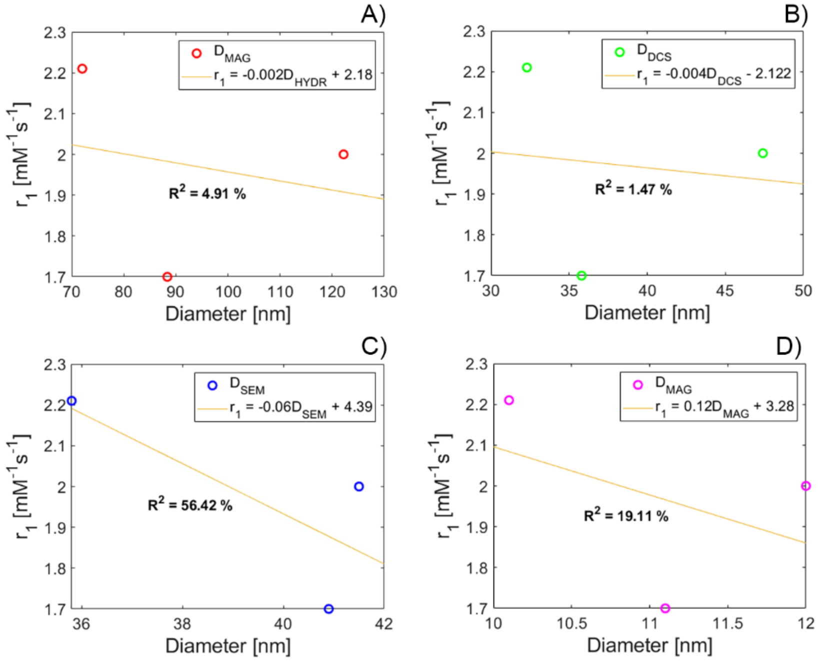

Figure S9. Linear regression analysis of the longitudinal relaxivity r_1 dependence on the diameter of DEX-coated MNPs determined by various techniques. D_{HYDR} (**A**), D_{DCS} (**B**), D_{SEM} (**C**), D_{MAG} (**D**)

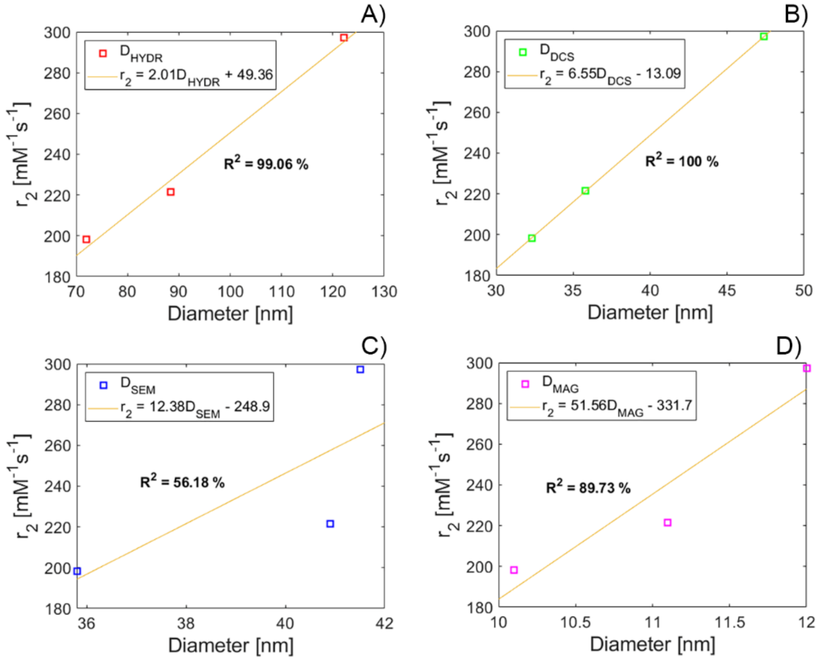

Figure S10. Linear regression analysis of the transversal relaxivity r_2 dependence on the diameter of DEX-coated MNPs determined by various techniques: D_{HYDR} (**A**), D_{DCS} (**B**), D_{SEM} (**C**), D_{MAG} (**D**)

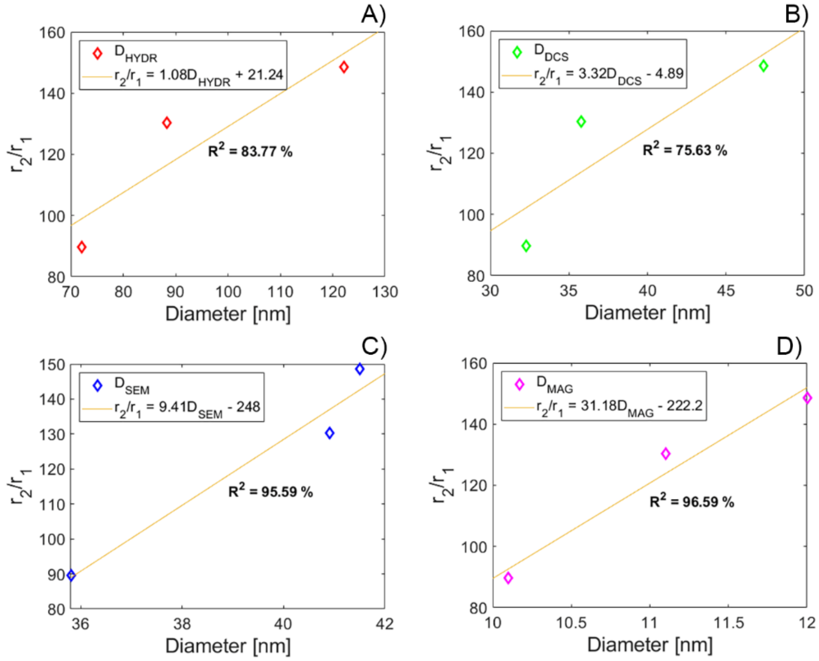

Figure S11. Linear regression analysis of the r_2/r_1 ratio dependence on the diameter of DEX-coated MNPs determined by various techniques: D_{HYDR} (**A**), D_{DCS} (**B**), D_{SEM} (**C**), D_{MAG} (**D**).
